# Identification of soybean seed developmental stage-specific and tissue-specific miRNA targets by degradome sequencing

**DOI:** 10.1186/1471-2164-13-310

**Published:** 2012-07-16

**Authors:** Md Shamimuzzaman, Lila Vodkin

**Affiliations:** 1 Department of Crop Sciences, University of Illinois, Urbana, IL, 61801, USA

## Abstract

**Background:**

MicroRNAs (miRNAs) regulate the expression of target genes by mediating gene silencing in both plants and animals. The miRNA targets have been extensively investigated in *Arabidopsis* and rice using computational prediction, experimental validation by overexpression in transgenic plants, and by degradome or PARE (parallel analysis of RNA ends) sequencing. However, miRNA targets mostly remain unknown in soybean (*Glycine max*). More specifically miRNA mediated gene regulation at different seed developmental stages in soybean is largely unexplored. In order to dissect miRNA guided gene regulation in soybean developing seeds, we performed a transcriptome-wide experimental method using degradome sequencing to directly detect cleaved miRNA targets.

**Results:**

In this study, degradome libraries were separately prepared from immature soybean cotyledons representing three stages of development and from seed coats of two stages. Sequencing and analysis of 10 to 40 million reads from each library resulted in identification of 183 different targets for 53 known soybean miRNAs. Among these, some were found only in the cotyledons representing cleavage by 25 miRNAs and others were found only in the seed coats reflecting cleavage by 12 miRNAs. A large number of targets for 16 miRNAs families were identified in both tissues irrespective of the stage. Interestingly, we identified more miRNA targets in the desiccating cotyledons of late seed maturation than in immature seed. We validated four different auxin response factor genes as targets for gma-miR160 via RNA ligase mediated 5’ rapid amplification of cDNA ends (RLM-5’RACE). Gene Ontology (GO) analysis indicated the involvement of miRNA target genes in various cellular processes during seed development.

**Conclusions:**

The miRNA targets in both the cotyledons and seed coats of several stages of soybean seed development have been elucidated by experimental evidence from comprehensive, high throughput sequencing of the enriched fragments resulting from miRNA-guided cleavage of messenger RNAs. Nearly 50% of the miRNA targets were transcription factors in pathways that are likely important in setting or maintaining the developmental program leading to high quality soybean seeds that are one of the dominant sources of protein and oil in world markets.

## Background

MicroRNAs (miRNAs) are endogenous noncoding small RNAs which play significant roles in the regulation of gene expression. Post-transcriptional gene regulation by miRNAs constitutes one of the most conserved and well characterized gene regulatory mechanisms. It is important for growth, development, stress responses and numerous other biological processes in eukaryotes [1-5]. Therefore, identification of miRNAs and their targets in diverse species has been a major focus in recent years [6,7]. In higher plants, miRNAs play significant roles in different developmental stages by regulating gene expression at transcriptional and post-transcriptional levels [8-12].

Most plant miRNAs facilitate the degradation of their mRNA targets by slicing precisely between the tenth and eleventh nucleotides (nt) from the 5’ end of the miRNA. As a result, the 3’ fragment of the target mRNA possesses a monophosphate at its 5’ end. This important property has been used to validate miRNA targets [8]. Isolation of such fragments is one of the critical steps for validating miRNA guided cleavage of target mRNA. A major limitation of this procedure is that every single predicted gene has to be verified separately. So, one-at-a-time isolation of cleaved RNA fragments is laborious, time-consuming and expensive. Recently, high-throughput sequencing methods, known as degradome analysis or PARE (parallel analysis of RNA ends) that can globally identify small RNA targets have been developed to overcome such limitations [13,14].

Soybean is one of the most important crops cultivated all over the world. It is a good source of vegetable protein and oil. However, the role of miRNAs in soybean seed development is mostly unknown. So it is important to identify the seed developmental stage-specific and tissues-specific miRNAs and their potential target genes. Identification of the consequences of miRNA-guided target degradation that occurs in a developmental and tissue specific manner could help to elucidate how lipid and protein metabolic pathways operated during seed development. The soybean genome (cv. Williams82) was decoded a year ago [15], and this information has accelerated molecular research on soybeans. Although many soybean miRNAs were identified in previous research [16-20], the number of miRNAs known in soybean is still very small and considerably lower than that in *Arabidopsis* or rice. High-throughput sequencing technologies such as massively parallel signature sequencing (MPSS), 454 and sequencing-by-synthesis (SBS) have enabled the identification of miRNAs in soybean. The extent of miRNA-directed post-transcriptional gene regulation in any organism can only be fully realized by identifying not only the miRNA component but also the set of their RNA targets.

Recently, miRNA targets have been reported for one of the many stages of soybean seed development, namely very early at 15 days after flowering, and without dissection of the maternal seed coats from the cotyledons which develop from the zygote [21]. To comprehensively investigate small RNA targets and provide basic information for further understanding of the miRNA-mediated post-transcriptional regulation during different soybean seed developmental stages, we constructed five separate degradome libraries derived from seed coats and cotyledons of different developmental stages representing the early, mid, and late maturation stages of seed development. The libraries were sequenced using SBS sequencing technology. The degradome dataset for the five different libraries was computationally analyzed. The majority of these reads mapped to the soybean transcriptome. A total of 183 target genes were confirmed as miRNA targets, which included both conserved and non-conserved miRNAs. Additionally, we have identified targets for 25 cotyledon-specific miRNAs, as well as 12 miRNAs and their potential targets found only in the seed coats. We found 16 miRNA families and their large number of targets that are found in both tissues. Moreover, we have validated Auxin Response Factors (ARFs) to be targets of gma-miR160, as verified by RNA ligase-mediated 5’ rapid amplification of cDNA ends (RLM 5’-RACE). The identification of developmental stage-specific and tissue-specific miRNA targets including many transcription factors advance our understanding of the miRNA-mediated post-transcriptional gene regulation during soybean seed development.

## Results

### Seed developmental stage-specific library construction, sequencing and sequence analysis

In higher plants, most miRNAs regulate their targets via cleavage, which normally occurs between the tenth and eleventh nucleotides of the complementary region between the miRNA and the mRNA target [22]. The 3’ cleavage fragments contain both a free 5’ monophosphate and a 3’ polyA tail. So, these cleavage products can be successfully ligated with RNA ligase, whereas full length cDNAs with a 5’ cap structure or other RNAs lacking the 5’ monophosphate group are not compatible for ligation [8] and thus will be unavailable for subsequent amplification and sequencing reactions.

Five different degradome libraries, which capture the cleaved mRNAs, were constructed from cotyledons and seed coats from different seed developmental stages. These represented early maturation seed (25–50 mg fresh weight, green seed) and mid-maturation (100–200 mg fresh weight, green seed), the stages when the biosynthetic capacity of the seed is maximal and proteins and oils are accumulated at a high rate. In addition, we constructed a library from the yellow cotyledons (300–400 mg fresh weight) that are undergoing dehydration and desiccation.

SBS sequencing of these libraries produced raw reads from 10 million to 45 million (Table 1). After removal of low quality sequences and adapter removal, 95% of these reads lengths were 20 or 21 nt in length as expected from the cloning procedure. More than 97% of reads mapped to the soybean genome available at the Phytozome data base [23]. We also used the computationally predicted cDNA transcripts from the soybean genome sequence consisting of 78,773 high and low confidence gene models (Glyma models) for mapping degradome reads and found that more than 95% of reads matched to the Glyma models. These data indicate our degradome libraries to be of high quality and efficiency in recovering degraded mRNA targets that should contain the sequence profile resulting from miRNA directed cleavage.

**Table 1:** Analysis of degradome reads from five different libraries matched to the soybean genome

**Library name**^**a**^	**Raw Reads**	**20-21 nt reads**	**Genome matched reads**^**b**^	**Transcriptome matched Reads**^**b**^
Cot25	27802207	25926963	25300772	24754628
SC25	41395866	40296710	39308067	37957708
Cot100	10473739	9088079	8902528	8646558
SC100	26326261	25931744	25289389	24436953
Cot300	44451740	41517379	40494429	40335132

^a^Cot25 and SC25 are libraries from cotyledons and seed coats dissected from early-maturation green seed of 25–50 mg fresh wt.; Cot100 and SC100 are cotyledons and seed coats dissected from mid-maturation green seed of 100–200 mg fresh weight range; Cot300 are cotyledons dissected from late maturation yellow seeds of 300–400 mg weight range.

^b^Genome matched reads indicate the number of reads of 20 or 21 nt that matched to *Glycine max* genome sequence available in Phytozome. Transcriptome matched reads indicate the number of reads that matched to *Glycine max* transcriptome (Glyma models) available in Phytozome [23].

### Systematic identification of miRNA targets in soybean

Systematic identification of miRNA targets was accomplished using previously described methods by analyzing the 20 and 21 nt reads with the CleaveLand pipeline for miRNA target identification [24] using all *Glycine max* miRNAs from miRBase [25,26]. The identified targets were grouped into five categories by the program based on the relative abundance of the number of reads mapping to the predicted miRNA target site relative to other sites in the gene model (see Methods for details). Those in category 0 clearly have the majority of tags located at the miRNA-guided cleavage site.

**Figure 1:**
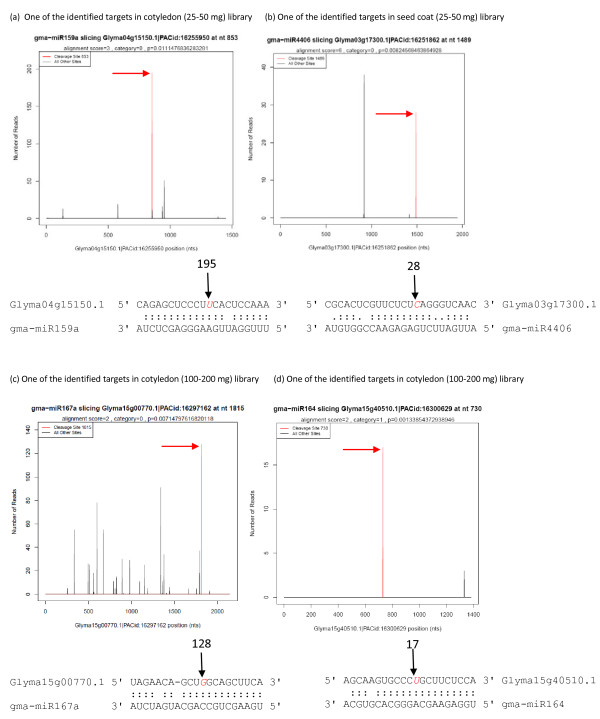
**Target plots (t-plots) of identified miRNA targets using degradome sequencing.** Representative t-plots (**a**-**d**) are shown, one from each of four different libraries of the cotyledon (25–50 mg fresh weight range), seed coat (25–50 mg), cotyledon (100–200 mg) and seed coat (100–200 mg). Red arrows indicate signatures consistent with miRNA-directed cleavage. mRNA:miRNA alignments along with the detected cleavage frequencies (absolute numbers) are shown above the black arrow. The red colored italicized nucleotide on the target transcript from the 3’ end indicates the cleavage site detected in the degradome library.

The identified miRNA targets using degradome sequencing are presented in the form of target plots (t-plots) that plot the abundance of the signatures relative to their position in the transcript [14]. Representative t-plots are shown, one from each of four different degradome libraries such as cotyledon (25–50 mg), seed coat (25–50 mg), cotyledon (100–200 mg) and seed coat (100–200 mg) (Figure 1). In each of the four Glyma models, a clear peak for the absolute number of tags is found at the predicted cleavage site for gma-miR159, gma-miR4406, gma-miR167, or gma-miR164.

The location of the cleavage site within the target gene is another important aspect of miRNA-mediated gene silencing. In soybean, the cleavage site of the miRNA was usually located in the CDS (coding sequence) of the target genes (Tables 2,3 and 4 and Additional file 1). Since the soybean genome at Phytozome [23] used computational predictions of gene models (known as Glyma models), some are likely deficient at the 5’ and 3’ UTRs (untranslated regions). Due to the some gene models being incomplete in the UTRs, there are likely other genes targeted by miRNA-guided cleavage in the UTR regions that may not be detected in our alignment analyses. In addition, miRNAs that function through translational repression, as opposed to cleavage of the target mRNA, will also not be identified by degradome or PARE sequencing techniques.

**Table 2:** Identified miRNA targets found in both soybean seed coat and cotyledon

**miRNA**	**Tissue**	**Target**	**Target Annotation**	**C.Site**	**Location**	**Category**	**TP100M**	**P-Value**
gma-miR156	C100	Glyma02g13370.1	SBP domain containing protein	1219	CDS	0	92.52237	0.0477
	C300	Glyma02g13370.1	SBP domain containing protein	1219	CDS	0	27.27151	0.0496
	SC100	Glyma02g30670.1	SBP domain containing protein	768	CDS	0	335.5574	0.0445
	C100	Glyma05g00200.1	SBP domain containing protein	1202	CDS	1	57.82648	0.015
gma-miR159*	**C25**	**Glyma04g15150.1**	**Myb family transcription factor**	**853**	**CDS**	**0**	**787.7315**	**0.0111**
	C300	Glyma04g15150.1	MYB family transcription factor	853	CDS	0	178.5044	0.0091
	C25	Glyma06g47000.1	Myb family transcription factor	852	CDS	0	787.7315	0.0111
	SC25	Glyma06g47000.1	Myb family transcription factor	852	CDS	0	18.44158	0.0123
	C300	Glyma06g47000.1	MYB family transcription factor	852	CDS	0	178.5044	0.0091
	SC100	Glyma13g34710.1	No functional annotation	1161	CDS	2	106.3962	0.0391
	C300	Glyma13g34710.1	No Functional Annotation	1161	CDS	1	9.916913	0.0133
	C25	Glyma19g40720.1	Anion Exchange protein	2162	CDS	1	28.27754	0.0157
gma-miR160^#^	C100	Glyma10g35480.1	Auxin response factor	740	CDS	0	12166.69	0.0168
	**C100**	**Glyma11g20490.1**	**Auxin response factor**	**1510**	**CDS**	**0**	**12166.69**	**0.0071**
	**C100**	**Glyma12g08110.1**	**Auxin response factor**	**1501**	**CDS**	**0**	**12166.69**	**0.0071**
	**C100**	**Glyma12g29720.1**	**Auxin response factor**	**1626**	**CDS**	**0**	**1445.662**	**0.0168**
	**C100**	**Glyma14g33730.1**	**Auxin response factor**	**1184**	**CDS**	**0**	**1144.964**	**0.027**
	SC25	Glyma04g43350.1	Auxin response factor	1337	CDS	0	1391.022	0.0379
	C300	Glyma04g43350.1	Auxin response factor	1337	CDS	0	401.635	0.0281
	SC25	Glyma10g06080.1	Auxin response factor	1355	CDS	0	821.9674	0.0379
gma-miR164*	**SC100**	**Glyma15g40510.1**	**No apical meristem protein**	**730**	**CDS**	**1**	**69.56677**	**0.0013**
	C100	Glyma04g33270.1	No apical meristem protein	634	CDS	0	150.3488	0.0026
	SC100	Glyma04g33270.1	No apical meristem protein	634	CDS	0	425.585	0.0024
	SC100	Glyma05g00930.1	No apical meristem protein	751	CDS	2	425.585	0.0433
gma-miR166	SC100	Glyma04g09000.1	START domain containing protein	93	CDS	3	16.36865	0.0478
	SC100	Glyma05g30000.1	HD-ZIP transcription factor	1041	CDS	3	8.184326	0.0478
	C25	Glyma05g30000.1	HD-ZIP transcription factor	1041	CDS	0	3389.265	0.0331
	C300	Glyma05g30000.1	HD-ZIP transcription factor	1041	CDS	0	4321.295	0.0271
gma-miR167*	**C100**	**Glyma15g00770.1**	**Zinc finger family protein**	**1815**	**CDS**	**0**	**1480.358**	**0.0071**
	SC100	Glyma02g18250.3	Elongation Factor S-II	90	CDS	1	3592.919	0.0168
	C100	Glyma02g40650.1	Auxin response factor	2924	CDS	0	855.8319	0.027
	SC100	Glyma02g40650.1	Auxin response factor	2924	CDS	0	1239.925	0.0252
	C25	Glyma02g40650.1	Auxin response factor	2924	CDS	0	2536.899	0.0343

Complete tables can be found in Additional file 1.

*CDS*: Coding Sequence; *UTR*: Untranslated Region; *TP100M*: Transcripts per 100 million; *C.site* (Cleavage site): Nucleotide number from 5’ end of cDNA; P-value ≤0.05 using Cleaveland pipeline.

Cot25 and SC25 are libraries from cotyledons and seed coats dissected from early-maturation green seed of 25–50 mg fresh wt.; Cot100 and SC100 are cotyledons and seed coats dissected from mid-maturation green seed of 100–200 mg fresh weight range; Cot300 are cotyledons dissected from late maturation yellow seeds of 300–400 mg weight range.

* and bold are miRNAs whose targets shown in the target plots (t-plots) (Figure 1).

# and bold are miRNAs whose RLM-5’RACE validated targets are shown in the target plots (t-plots) (Figure 2).

**Table 3:** Potential miRNA targets found only in cotyledons at different seed developmental stages

**miRNA**	**Tissue**	**Target**	**Target Annotation**	**C.Site**	**Location**	**Category**	**TP100M**	**P-Value**
gma-miR171	C25	Glyma15g01820.1	Protein tyrosine kinase	1359	CDS	1	24.2379	0.0013
	C300	Glyma12g08490.1	Putative methyltransferase	434	CDS	1	12.3961	0.0462
gma-miR394	C100	Glyma06g13230.3	Ferredoxin related protein	1070	CDS	1	104.088	0.027
	C100	Glyma06g13230.2	Ferredoxin related protein	685	CDS	1	104.088	0.027
	C100	Glyma14g25810.1	GTPase-activating protein	1013	CDS	1	57.8265	0.0035
	C100	Glyma06g13230.1	Ferredoxin related protein	952	CDS	1	104.088	0.027
gma-miR398	C100	Glyma03g40280.2	Copper/Zinc superoxide dismutase	156	5' UTR	2	57.8265	0.0379
	C100	Glyma03g40280.3	Copper/Zinc superoxide dismutase	156	5' UTR	2	57.8265	0.0379
gma-miR1509	C300	Glyma18g03980.2	No Functional Annotation	2155	3' UTR	0	7.43768	0.0041
gma-miR1513	C300	Glyma08g27950.1	F-box domain containing protein	190	CDS	0	29.7507	0.0402
gma-miR1514	C25	Glyma07g05360.2	No apical meristem protein	773	CDS	0	16.1586	0.0371
gma-miR1515	C300	Glyma09g02920.1	PAZ domain containing protein	2750	CDS	0	57.0223	0.003
gma-miR1531	C100	Glyma16g33400.1	Serine protease inhibitor family	536	3' UTR	3	46.2612	0.0414
gma-miR1532	C25	Glyma10g28900.1	Universal stress protein family	626	3' UTR	4	4.03965	0.015
gma-miR1535	C25	Glyma08g22920.1	Ribose 5-phosphate isomerase	168	CDS	1	60.5947	0.0242
gma-miR2109	C25	Glyma03g14900.1	LRR containing protein	46	CDS	0	88.8723	0.0165
gma-miR4357	C300	Glyma13g01500.1	Alg9-like mannosyltransferase	779	CDS	1	22.3131	0.0089
	C300	Glyma01g35530.1	Transferase family protein	522	CDS	1	9.91691	0.0114
gma-miR4369	C25	Glyma19g43800.1	Calcineurin-like phosphoesterase	1257	3' UTR	4	4.03965	0.0468
gma-miR4371	C300	Glyma15g08400.4	No Functional Annotation	452	3' UTR	0	17.3546	0.0017
gma-miR4380	C300	Glyma06g10840.1	MYB family transcription factor	630	CDS	1	9.91691	0.0347
gma-miR4387	C300	Glyma08g43670.1	Uncharacterized conserved protein	1501	CDS	0	4.95846	0.0068
gma-miR4390	C100	Glyma15g06380.1	Dynamin family protein	287	CDS	3	104.088	0.0251
gma-miR4398	C25	Glyma02g25150.1	Integrase domain containing protein	199	5' UTR	4	4.03965	0.0444
gma-miR4402	C300	Glyma02g18090.1	Lectin domain containing protein	783	CDS	4	2.47923	0.0293
gma-miR4403	C300	Glyma19g36500.1	No Functional Annotation	1860	3' UTR	4	2.47923	0.0484
gma-miR4408	C25	Glyma16g34800.1	No functional annotation	434	CDS	1	68.674	0.0057
gma-miR4409	C25	Glyma16g04060.3	BTB/POZ domain containing protein	1304	3' UTR	0	12.1189	0.0144
gma-miR4415	C100	Glyma02g20490.2	Transferase family protein	502	CDS	1	57.8265	0.0463
	C100	Glyma02g20490.1	Transferase family protein	502	CDS	1	57.8265	0.0463
	C100	Glyma15g12430.1	Transferase family protein	464	CDS	1	57.8265	0.0463
gma-miR4416	C100	Glyma01g44970.1	BTB/POZ domain containing protein	434	CDS	0	161.914	0.0318

*CDS*: Coding Sequence; *UTR*: Untranslated Region; *TP100M*: Transcripts per 100 million; *C.site* (Cleavage site): Nucleotide number from 5’ end of cDNA; P-value ≤0.05 using Cleaveland pipeline.

**Table 4:** Potential miRNAs and their targets found only in seed coats at different developmental stages

**miRNA**	**Tissue**	**Target**	**Target Annotation**	**C.Site**	**Location**	**Category**	**TP100M**	**P-Value**
gma-miR319	SC100	Glyma12g33640.1	TCP family transcription factor	740	CDS	0	106.396	0.0231
	SC100	Glyma15g09910.1	TCP family transcription factor	959	CDS	0	69.5668	0.0121
gma-miR393	SC100	Glyma19g27280.1	LRR containing protein	2207	CDS	2	20.4608	0.032
	SC100	Glyma03g36770.1	LRR containing protein	1750	CDS	3	167.779	0.031
	SC100	Glyma16g05500.1	LRR containing protein	2279	CDS	3	20.4608	0.0132
	SC100	Glyma19g39420.1	LRR containing protein	1751	CDS	3	167.779	0.031
gma-miR1508	SC100	Glyma13g35890.1	EF-hand containing protein	517	3' UTR	1	8.18433	0.0385
gma-miR1518	SC100	Glyma15g12180.1	Ubiquitin-protein ligase	273	CDS	1	16.3687	0.02
gma-miR1523	SC25	Glyma20g02470.1	LRR containg protein	407	CDS	1	5.26902	0.0419
gma-miR1526	SC100	Glyma08g09640.1	No Functional Annotation	424	CDS	0	163.687	0.048
	SC100	Glyma08g09640.2	No Functional Annotation	424	CDS	0	163.687	0.048
gma-miR2119	SC100	Glyma14g24860.1	Alcohol dehydrogenase	99	CDS	2	2488.04	0.0462
gma-miR4374	SC100	Glyma01g36660.1	Ankyrin repeat containing protein	2124	3' UTR	1	8.18433	0.009
gma-miR4377	SC25	Glyma06g04910.1	Cyclin family protein	910	CDS	4	2.63451	0.0297
gma-miR4399	SC100	Glyma17g34370.1	No functional annotation	637	3' UTR	4	4.09216	0.0491
gma-miR4406	SC25	Glyma03g17300.1	Glyoxal oxidase related protein	1489	CDS	0	73.7663	0.0082
gma-miR4407	SC100	Glyma08g15670.1	Peptide transporter family protein	1078	CDS	1	1076.24	0.0159
	SC100	Glyma05g04810.1	Peptide transporter family protein	918	CDS	1	1076.24	0.0159

*CDS*: Coding Sequence; *UTR*: Untranslated Region; *TP100M*: Transcripts per 100 million; *C.site* (Cleavage site): Nucleotide number from 5’ end of cDNA; P-value ≤0.05 using Cleaveland pipeline.

* and bold is miRNA whose target is shown in the target plot (t-plot) (Figure 1).

The full complement of targets found in each of the five degradome libraries is presented in Additional file 1. In total, 183 targets representing 53 different miRNAs families were identified. Of those 133 targets were found representing the putative action of 16 different miRNAs in common between both tissues. Table 2 presents a subset of those that are found in at least one stage of development for both seed coats and cotyledons. The CleaveLand program predicts any gene family members that have a splice site matching the degradome data. Some miRNA family members residing at different genomic locations have very similar, if not identical mature miRNA sequences. Thus, the predictions from analysis of degradome data do not necessarily mean that the particular miRNA family member revealed from degradome data is the one expressed in that tissue. Direct sequencing of the small RNA population is required to verify the presence of a particular gene family member. Inspection of small RNA sequencing data from seed coats and cotyledons of Williams [27] shows the presence of various miRNA family members for gma-miR156, 159, 160, 164, 166, and 167, thus confirming that these miRNAs are present during seed development.

### Identification of miRNA targets specific to either seed coat or cotyledons during seed development

Tissue specific miRNA and target identification is very important for understanding the regulation of gene expression in a spatial manner. In this study, we constructed cotyledon and seed coat libraries separately to identify miRNA targets both at younger (25–50 mg fresh weight seed) and older stages (100–200 mg and 300–400 mg fresh weight seed) of soybean seed development. Tissue specific siRNAs generated from a cluster of inverted repeat chalcone synthase (CHS) genes that downregulate CHS mRNAs and lead to lack of pigment on soybean seed coats have been described [27,28], but very little is known about the miRNAs and their targets in developing seed tissues. We analyzed the degradome data from seed coats versus cotyledons and identified 25 miRNAs and their 32 different targets that were found only in the cotyledons and not the seed coats (Table 3). Likewise, 12 miRNAs and their 18 targets are associated with the seed coats only (Table 4).

### Validation of miRNA targets

**Figure 2:**
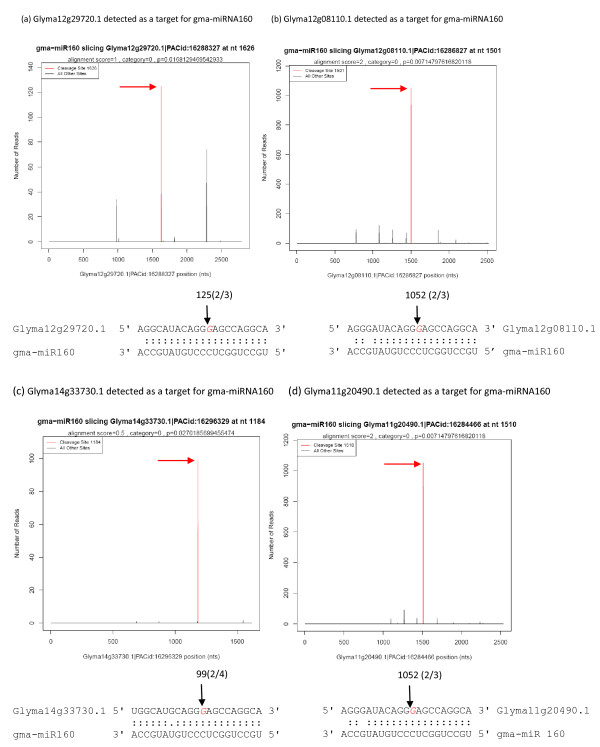
**Validation of Auxin Response Factors (ARFs) regulated by gma-miR160 in cotyledon.** Confirmed targets (**a**-**d**) for gma-miR160 are presented in the form of target plots (t-plot) and alignments. Absolute numbers of signature sequences are indicated in the t-plot. Red arrows indicate signatures consistent with miRNA-directed cleavage. The black arrows indicate a site verified by RLM 5’-RACE and detected cleavage frequencies (absolute numbers) are shown above the arrow. The cleavage site is shown as a red letter. Cleavage frequency as determined by gene-specific 5’-RACE at the indicated position is shown in parenthesis.

We report here that many targets were captured by the degradome analysis, which provided experimental evidence to support previous computational predictions. Because of its polyploid genome, many soybean genes are present in multiple copies. As a result, some of the reads align to multiple members of the same gene family. To further confirm the degradome data for some of the family members, a RLM-5’ RACE experiment was performed to examine which family members were targeted by the miRNA for degradation. For gma-miR160 in the cotyledon (100–200 mg) degradome library (Table 2 and Additional file 1), we have identified five targets annotated as Auxin Response Factors (ARFs). Four of the five, namely *Glyma12g08110.1*, *Glyma12g29720.1*, *Glyma14g33730.1* and *Glyma11g20490.1,* were also verified by RLM-5’RACE to be subjected to cleavage guided by gma-miR160 (Figure 2).

### GO analysis of miRNA target genes in soybean seed developmental stages

**Figure 3:**
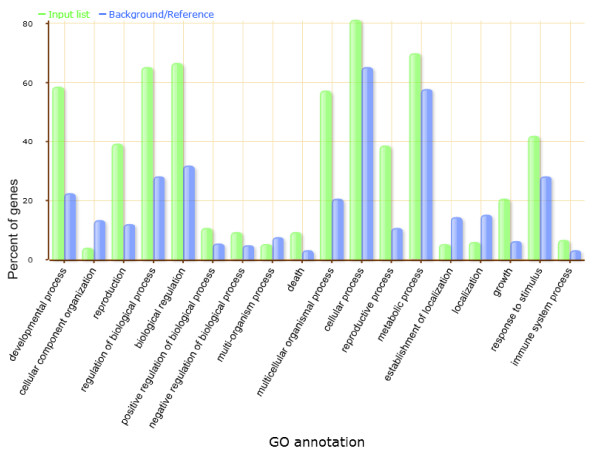
**GO analysis of miRNA target genes identified in cotyledons in different soybean seed developmental stages.** Green bars indicate the enrichment of miRNA targets in GO terms. Blue bars indicate the percentage of total annotated soybean genes mapping to GO terms. Only the predicted target genes for miRNAs identified by degradome sequencing were considered.

The identified targets for miRNAs in the three cotyledon degradome libraries were classified by their gene ontology (GO) using the AgriGO toolkit [29] (Figure 3). Higher percentages of these targets were found to be involved in developmental, reproductive, and regulatory and metabolic processes with respect to their proportions within the GO classification of all soybean cDNAs. The same general pattern is found for the targets predicted with the seed coats ( Additional file 2). The enrichment of the genes involved in developmental and regulatory processes may be consistent with the fact that the degradome libraries were constructed from different stages of developing soybean seeds. For the developing seeds, it is of utmost important to accumulate proteins and lipids that are subsequently used as the source of energy and amino acids for the germinating seedling. The corresponding miRNAs may regulate the expression of these target genes during different seed developmental stages in soybean through affecting various transcription factors that induce or shut off specific metabolic networks during the course of seed development. Interestingly, we identified more miRNA targets in the cotyledons of late seed maturation than earlier stages with a total of 92 different targets in the 300–400 desiccating, yellow seeds compared to 60 and 53 total in the early and mid-maturation, immature green seed respectively.

## Discussion

Regulation of gene expression by miRNAs has been comprehensively investigated in animals and plants [3,4,22,30,31]. In the case of higher plants, *Arabidopsis* and rice miRNA targets have been widely studied by high throughput sequencing [13,32-34]. Soybean is a polyploid crop plant having a complex and large genome compared to *Arabidopsis* and rice. The number of identified miRNAs and their potential targets in soybean is limited. To date, degradome sequencing has been reported for only one soybean tissue, namely the very young whole seed extracted 15 days after flowering from the cultivar Heinong44 [21]. In order to study the regulation of gene expression during soybean seed development, we constructed and sequenced five distinct degradome libraries using cotyledons and seed coats as a tissue source from different seed developmental stages of the cultivar Williams. The seed coat is primarily maternal tissue while the cotyledons represent the embryo of the next generation. Both tissues have the same genotype in inbred soybean lines.

After computational analysis using the Cleaveland pipeline [24], we identified a total of 183 potential targets of 53 miRNA families in five different seed developmental stage specific degradome libraries. Subsequent analysis and identification of cotyledon and seed coat specific miRNAs and their targets give us a better understanding of the regulation of gene expression in a spatial manner during soybean seed development at later stages of seed development in a widely used Maturity Group III cultivar “Williams”. The soybean genome sequence and predicted gene models used in this study are derived from a closely related isoline Williams82 [15]. Validation of four Auxin Response Factor (ARF) genes as targets of gma-miR160 indicates degradome sequencing as an efficient strategy to identify miRNA targets in plants.

Comparison of the data in our report with the first soybean degradome data reported by Song et al. [21] from a very early stage of soybean seed development in the cultivar Heinong44 revealed a number of miRNA targets in common among the data sets including many transcription factors such as ARF, MYB, TCP, NF-Y, Growth Regulatory Factor, HD-ZIP, PPR, SBP and NAC family protein. In contrast, we found some other miRNA targets such as Permease family protein, LRR domain containing protein, transmembrane protein 14 C, Serine-Threonine kinase, BRE expressed protein and transcription factor TFIID only in our degradome libraries that represented later stages of seed development of the cultivar Williams. Overall there were 65 Glyma model targets in common between both of the data sets, 80 found only in the Heinong44 data set, and 118 found only in the Williams data sets.

The 183 identified targets from our degradome analyses belonged to 57 different annotation groupings ( Additional file 1). Comparing those to the Song et al. paper, 11 annotation groups are in common, 18 are unique to the Heinong44 very young seed, and 46 are unique to the five libraries of the Williams data sets (Table 5). Many of the identified soybean miRNA targets belong to diverse gene families of transcription factors such as ARFs, MYBs, TCPs, NACs, HD-ZIPs and NF-Y subunits (Tables 2,3 and 4 and Additional file 1). Many of these transcription factors are known to regulate diverse aspects of plant growth and development. Patterning and outgrowth of lateral organs in plants depend on the expression of HD-ZIP transcription factors that specify adaxial/upper cell fate [35,36]. We identified a number of HD-ZIP transcription factors as targets for gma-miR166 in our degradome libraries. MYB family members in rice, which are targeted by miR159, appear to play an important role in response to the presence of abscisic acid (ABA) during plant embryonic development, suggesting their roles in seed development [37,38]. In this study, we detected a number of MYB family transcription factors regulated by gma-miR159 (Additional file 1. The gma-miRNA156 family members target sites in numerous proteins containing the Squamosa Promoter Binding (SBP) domain. SBP and SBP-LIKE (SPLs) proteins play multiple roles in plant development [39,40]. In *Arabidopsis*, rice and in some other plants, miR156 regulates leaf development by targeting Squamosa-Promoter Binding protein-like (SBP) transcription factors [31,39]. The identification of SBP as a target of gma-miR160 may indicate the additional level of regulation for SBP during soybean seed development.

**Table 5:** Comparison of miRNA target annotations between the Williams and Heinong44 data sets

**Annotations Unique to Williams**	**Annotations Unique to Heinong44**	**Annotations Found in Both Data Sets**
Alcohol/Zinc-binding dehydrogenase	Polyubiquitin protein	AGO protein
Alg9-like mannosyltransferase	Plasmamembrane protein	Auxin Response Factor
Aluminium induced protein	Auxin signaling F-BOX protein	Copper/zinc superoxide dismutase
Anion Exchange protein	NADP+	Growth Regulating Factor
Ankyrin repeat containing protein	MtN19-like protein	HD-ZIP Transcription Factor
Ataxin-2 domain containing protein	Serine-type endopeptidase	MYB family Transcription Factor
bHLH family protein	elongation factor	No Apical Meristem protein (NAC Family)
BRE Expressed protein	NSF attachment protein	SBP domain containing protein
BTB/POZ domain containing protein	Autophagy protein	TCP family transcription factor
Calcineurin-like phosphoesterase	embryo-related protein	Nuclear Factor-YA
Cellulose synthase	AP2 transcription factor	Zinc Finger Family protein
Cyclin family protein	heat shock cognate protein	
Dynamin family protein	expressed protein	
EF-hand containing protein	60 S ribosomal protein	
Elongation Factor S-II	Disulfide isomerase	
F-box domain containing protein	FAD linked oxidase family protein	
Ferredoxin related protein	Auxin inducible transcription factor	
Glyoxal oxidase related protein	ribulose-1,5-bisphosphate carboxylase	
GTPase-activating protein		
Integrase domain containing protein		
lectin domain containing protein		
LRR containing protein		
mRNA capping enzyme		
NADP/FAD oxidoreductase		
NB-ARC domain containing protein		
No Functional Annotation		
PAZ domain containing protein		
Peptide transporter family protein		
Permease family protein		
PPR repeat containing protein		
Protein tyrosine kinase		
Putative methyltransferase		
Ras family protein		
Ribose 5-phosphate isomerase		
Serine protease inhibitor family		
Serine-threonine protein kinase		
START Domain containing protein		
TIR domain containing protein		
Transcription factor TFIID		
Transferase family protein		
Transmembrane protein 14 C		
Transporter family protein		
UBA domain containing protein		
Ubiquitin-protein ligase		
Uncharacterized conserved protein		
Universal stress protein family		

Williams data sets from this study were generated using five degradome libraries of early, mid and late soybean seed developmental stage. The Heinong44 data set represented an earlier seed stage [21].

Our analyses of the early, mid and late maturation developmental stages of soybean show a number of targets similar to those found by Song et al., [21] in the very young seeds of the cultivar Heinong44 including the SPB transcription factors. One notable difference was the absence in our degradome data of miRNA172 targets which include members of the AP2 transcription factor family. From inspection of sequenced small RNA populations from the 50–75 mg seed coats and cotyledons of Williams [27], we find only a few occurrences of the miR172 family (less than 30 occurrences per million reads) while some family members of the miR156 family are highly abundant (99,000 per million) in the cotyledons. We speculate that miR172 and/or its targets may be more abundant in the very young seed used by the Song et al. group [21] and not prevalent in the mid-maturation seed that we have examined. In *Arabidopsis*, miR172 has been reported to be involved in the regulation of flowering time and floral development [40]. Alternatively, the AP2 factors may not be detected as targets in the degradome data if translational repression by miR172 is operative as has been shown in *Arabidopsis* flower development [41]. Nuclear Factor Y (NF-Y) was shown to control a variety of agronomically important traits, including drought tolerance, flowering time, and seed development [42]. We detected seven NF transcription factor YA subunit mRNAs specifically in seed coats that are targets of miR169 family members that occur in both seed coats and cotyledons ( Additional file 1). These targets may indicate some specific regulation of NF-YA transcription factors during soybean seed development.

To obtain a deeper understanding of soybean seed development, we investigated tissue specific miRNA target identification in the cotyledons and seed coats at different seed developmental stages. Based on the degradome data, we identified some miRNAs that may act differentially in the cotyledons versus the seed coats to degrade their targets (Tables 3 and 4). F-box proteins involved in auxin-stimulated protein degradation (TIR1-like) were among the identified targets specifically found in soybean cotyledon (Table 3). The LRR kinases have been reported to play important roles in plant development and brassinosteroid and ABA signaling [43]. We identified several LRR domain containing proteins as targets for gma-miR393, gma-miR1523 and gma-miR2109 (Tables 2,3 and 4). The presence of these miRNA targets implies their regulation during soybean seed development. As a storage organ, the soybean seed contains significant amounts of lipid and protein. Thus the regulation of energy metabolism is very important during seed development. We identified a number of targets in the soybean cotyledon such as NADP/FAD oxidoreductase, ribose-5-phosphate isomerase, GTPase activating proteins and ferredoxin related proteins which are related to energy metabolism. Both in the soybean cotyledon and seed coat, we found pentatricopeptide repeat (PPR) proteins as targets of miR1520 (Table 2 and Additional file 1) which regulates gene expression in the mitochondria and chloroplasts [44,45]. Since we constructed our degradome libraries using cotyledons and seed coats from different seed developmental stages, we identified targets of miRNAs during a broad range of soybean seed development.

Auxin is an important phytohormone in higher plants. It acts as a key player in plant development [46]. As the transducer of auxin signaling, ARFs play vital roles in plant development, including shoot, root and flower formation [47,48]. In *Arabidopsis*, miR160 and miR167 are involved in auxin signaling via regulation of ARF genes [1]. In rice, a number of ARF encoding genes have been identified which are regulated by osa-miR160 and osa-miR167, respectively [33,34]. In our study, we identified a large number of ARF genes as targets for different miRNAs such as gma-miR160 and miR167. In the cotyledon (100–200 mg) degradome library ( Additional file 1), we identified five targets annotated as Auxin Response Factors (ARFs) for gma-miR160, and four of these, *Glyma12g08110.1Glyma12g29720.1Glyma14g33730.1* and *Glyma11g20490.1,* were validated by RLM-5’RACE showing precise cleavage as expected (Figure 2). These results suggested that gma-miR160 could participate in auxin signaling via down-regulation of ARFs during soybean seed developmental stages. The cleaved mRNAs captured by the degradome procedure indicate that the levels of the ARF mRNA targets are likely to be decreased, but qRTPCR or RNA sequencing data would be needed to directly confirm the effect on mRNA levels for a particular ARF target gene.

In our degradome libraries, miRNA targets are involved in major transitions between each stage of seed development and transcription factors account for approximately half of these targets. Of 183 identified targets in our soybean degradome libraries, GO analysis for biological function indicates that these genes are mainly involved in developmental and metabolic processes (Figure 3 and Additional file 2). Enrichment of developmentally related genes as target miRNAs suggests the high level of regulation of gene expression during soybean seed development. The larger number of targets found in the 300–400 mg desiccating, yellow cotyledons of late maturation implies that post-transcriptional regulation by miRNAs may aid in shifting the developmental program of the immature soybean cotyledons from biosynthesis of storage reserves to a catabolic role in utilization of those reserves during seed germination and growth. The miRNA targets verified by degradome sequencing will provide useful information for understanding and revealing significant roles of miRNAs during soybean seed development.

## Conclusion

Degradome sequencing is a valuable tool for the experimental confirmation of miRNA targets in higher plants. This method can reveal additional targets which are difficult to identify by computational prediction alone and confirm that the targets genes have been cleaved in specific tissues. Five degradome libraries from three different developmental stages identified 183 miRNA targets. Identification of soybean seed coat and cotyledon specific miRNA targets gives better understanding of tissue specific miRNA targets during seed development. In summary, the current study has confirmed a large set of targets that are subjected to miRNA guided degradation including many transcription factors and a surprisingly large number of targets in the late stages of cotyledon development. The data provides an avenue to explore more details about developmental stage specific miRNA targets that play critical roles in each of the important tissues during seed development.

## Methods

### Plant materials

Soybean (*Glycine max* cv. Williams) plants were grown in a greenhouse and seeds were collected at different developmental stages including early maturation, green 25–50 mg fresh weight seed, mid- maturation green 100–200 mg, and late maturation yellow 300–400 mg fresh weight seed. Immediately, cotyledons and seed coats were separated by dissecting whole seeds and then frozen in liquid nitrogen. Subsequently the tissue was freeze dried and stored at −80°C.

### RNA extraction and degradome library construction

Total RNA was extracted from freeze dried cotyledons and seed coats using a modified McCarty method [49] using phenol-chloroform extraction and lithium chloride precipitation. Approximately 200 μg of total RNA was used to select poly (A) RNA using the Oligotex mRNA mini kit (Qiagen). The degradome libraries were constructed as previously described [14,50]. Briefly, using T4 RNA ligase (Ambion), a 5’ RNA adapter (5’-GUUCAGAGUUCUACAGUCCGAC-3’, Dharmacon Inc.) was added to the cleavage products, which possess a free 5’-monophosphate at their 3’ termini. Then the ligated products were purified by Oligotex mRNA mini kit (Qiagen) and reverse transcribed using an oligo dT primer (5’-CGAGCACAGAATTAATACGACTTTTTTTTTTTTTTTTTTV-3’, Integrated DNA Technologies) via SuperScript II RT (Invitrogen). The generated cDNA was amplified for 6 cycles (94°C for 30 s, 60°C for 20 s, and 72°C for 3 min) with a pair of primers (forward, 5´-GTTCAGAGTTCTACAGTCCGAC-3´and reverse, 5´-CGAGCACAGAATTAATACGACT-3´, Integrated DNA Technologies) using Phusion Taq (New England Biolabs). The PCR products were digested with restriction enzyme Mme I (NEB). Next, a double stranded DNA adapter (top 5’-p- TGGAATTCTCGGGTGCCAAGG-3’, bottom 5’ –CCTT GGCACCCGAGAATTCCANN-3’, Integrated DNA Technologies) was ligated to the digested products using T4 DNA ligase [13]. Then the ligated products were selected based on size by running 10% polyacrylamide gel. The gel-purified products were used for the final PCR amplification (94°C for 30 s, 60°C for 20 s, and 72°C for 20 s) with primers (forward, 5’-AATGATACGGCGACCACCGAGATCTACACGTTCAGAGTTCTACAGTCCGA-3’, reverse, 5’-CAAGCAGAAG ACGGCATACGAGATCGTGATGTGACTGGAGTTCCTTGGCACCCGAGAATTCCA-3’, Integrated DNA Technologies) for 20 cycles. Finally, PCR products were gel purified and subjected to SBS sequencing by the Illumina HiSeq2000 at the Keck Center, University of Illinois at Urbana-Champaign. The sequencing data of the five degradome libraries are available under NCBI-GEO [51] series accession no. GSE34433.

### Initial processing and analysis of reads for different sequencing libraries

Degradome libraries were sequenced by the Illimuna HiSeq2000. The raw data were preprocessed to remove low quality reads and clip adapter sequences. Subsequently only 20–21 nt sequences with high quality scores were collected for analysis. The ultrafast Bowtie aligner [52] was used to map soybean degradome reads to the Phytozome *Glycine max* gene models [23]. The distinct reads that perfectly matched soybean transcript sequences remained. The CleaveLand pipeline [24] was used to find sliced miRNA targets using the Phytozome *Glycine max* gene models [23] and all *Glycine max* miRBase, release 17 [25,26] containing 207 mature miRNA sequences as input. All alignments with scores up to 7 and no mismatches at the cleavage site (between the tenth and eleventh nucleotides) were considered candidate targets. This analysis was performed separately for all five libraries. The identified targets were grouped into five categories based on the relative abundance of the degradome signatures at the miRNA target sites as determined by the program that indicates the abundance of the fragments mapping at the predicted miRNA target site relative to the abundance of fragments found at other sites. In category 0, the most abundant tags are found at the predicted site of miRNA guided cleavage and there was only one maximum on the transcript. If there was more than one abundant tag, it is indicated as category 1. In category 2, the abundance of cleavage signatures was less than the maximum but higher than the median. It was grouped as category 3 when the abundance of cleavage signatures was equal to, or less than the median. Very low abundance signatures including only 1 read was categorized as category 4.

### RLM 5’-RACE

For mapping the cleavage site within the miRNA target, a modified procedure for RNA ligase mediated (RLM-5’ RACE) was performed using the FirstChoice RLM-RACE Kit (Ambion). Total RNA was isolated from cotyledons and seed coats separately at different soybean seed developmental stages. Poly (A) + RNAs were purified using Oligotex mRNA mini kit (Qiagen). A 5’ RNA adaptor (5'-GCUGAUGGCGAUG AAUGAACACUGCGUUUGCUGGCUUUGAUGAAA- 3', Ambion RACE kit) was ligated to approximately 100 ng of mRNA using T4 RNA ligase. The ligated mRNAs were then reverse transcribed using oligo(dT) primer via M-MLV reverse transcriptase (Ambion). Two rounds of 5’ RACE reactions were performed with two nested primers (outer, 5'-GCTGATGGCGATGAATGAACACTG-3’; inner, 5'-CGCGGATCCGAAC ACTGCGTTTGCTGGCTTTGATG-3’, Ambion) and two gene-specific outer and inner primers (Additional file 3). Subsequently PCR products were gel purified and sequenced.

## Competing interests

The authors declare that they have no competing interests.

## Authors’ contributions

MS designed experiments, performed RNA extractions, constructed degradome libraries, analyzed degradome data and drafted the manuscript. LOV designed initial approach, obtained funding, led and coordinated the project, and edited the manuscript. All authors have read and approved the final manuscript.

## Authors’ information

Department of Crop Sciences, University of Illinois, Urbana, Illinois, 61801, USA.

## Supplementary Material

Additional file 1 Complete list of identified miRNA targets identified in soybean seed coats and cotyledons during seed development. click here for file

Additional file 2 GO analysis of miRNA targets identified in seed coats in different soybean seed developmental stages. Click here for file

Additional file 3 Gene Specific primer sequences for RLM-5’RACE. click here for file
