# Supplementary figures and images for: Identification of soybean seed developmental stage-specific and tissue-specific miRNA targets by degradome sequencing
Source: BMC Genomics. 2012 Jul 16;13:310. doi: 10.1186/1471-2164-13-310 (PMC3410764; doi:10.1186/1471-2164-13-310)

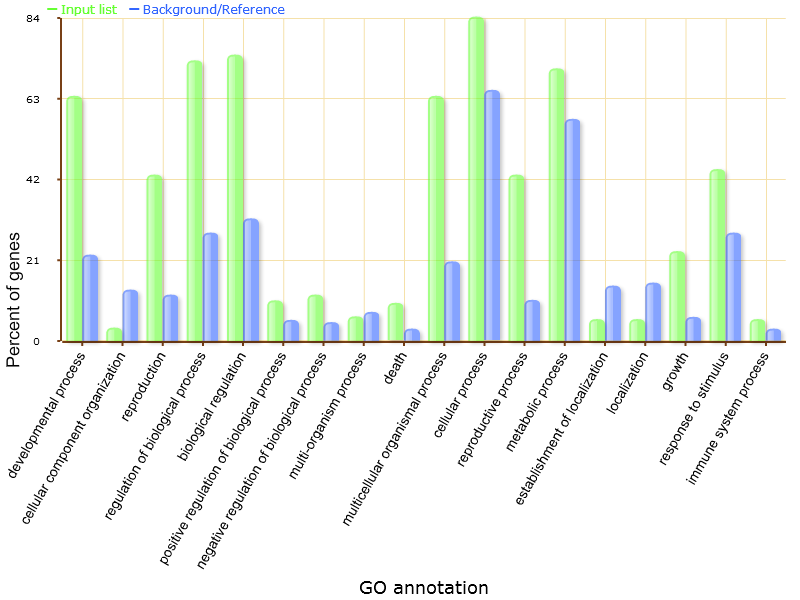

Supplement: Additional file 2 — GO analysis of miRNA targets identified in seed coats in different soybean seed developmental stages. [file 1471-2164-13-310-S2.doc]
